# Bibliometric and Visual Analysis on Metabolomics in Coronary Artery Disease Research

**DOI:** 10.3389/fcvm.2022.804463

**Published:** 2022-03-25

**Authors:** Ning Yu, Ruirui Wang, Baocheng Liu, Lei Zhang

**Affiliations:** Shanghai Innovation Center of TCM Health Service, Shanghai University of Traditional Chinese Medicine, Shanghai, China

**Keywords:** VOSviewer, CiteSpace, metabolomics, coronary artery disease, visual analytics

## Abstract

**Background:**

Metabolomics has immense research value in coronary artery disease and has drawn increasing attention over the past decades. Many articles have been published in this field, which may challenge researchers aiming to investigate all the available information. However, bibliometrics can provide deep insights into this research field.

**Objective:**

We aimed to qualitatively and quantitatively study metabolomics and coronary artery disease research, visually analyse the development status, trends, research hotspots, and frontiers of this field, and provide a reference for research on coronary artery disease.

**Methods:**

Articles were acquired from the Web of Science Core Collection. VOSviewer and CiteSpace software were used to analyse publication growth, country/region, institution, journal distribution, author, reference, and keywords, and detected the keywords with strong citation burstness to identify emerging topics.

**Results:**

A total of 1121 references were obtained, and the annual number of publications increased over the past 16 years. Metabolomics research has shown a gradual upward trend in coronary artery disease. The United States of America and China ranked at the top in terms of percentage of articles. The institution with the highest number of research publications in this field was Harvard University, followed by the University of California System and Brigham Women’s Hospital. The most frequently cited authors included Hazen SL, Tang WH, and Wang ZN. Ala-Korpela M was the most productive author, followed by Clish CB and Adamski J. The journal with the most publications in this field was Scientific Reports, followed by PLoS One and the Journal of Proteome Research. The keywords used at a high frequency were “risk,” “biomarkers,” “insulin resistance,” and “atherosclerosis.” Burst detection analysis of top keywords showed that “microbiota,” “tryptophan,” and “diabetes” are the current research frontiers in this field.

**Conclusion:**

This study provides useful information for acquiring knowledge on metabolomics and coronary artery diseases. Metabolomics research has shown a gradual upward trend in coronary artery disease studies over the past 16 years. Research on tryptophan metabolism regulated by intestinal flora will become an emerging academic trend in this field, which can offer guidance for more extensive and in-depth studies in the future.

## Introduction

Coronary artery disease (CAD) is the most common form of cardiovascular disease (CVD) and remains the leading cause of mortality and morbidity worldwide (1). Despite the several risk managements strategies, preventive measures, and treatment modalities that have been initiated, patients continue to die from cardiac-related complications. Hence, there is a need to identify novel therapeutic strategies for managing this condition (2). Although angiography is the gold standard for CAD diagnosis, it is highly invasive. Identification of circulating biomarkers provides a rapid, effective, and less invasive tool and may help identify CAD risk early in diagnosing and guiding treatment (3).

Metabolomics and metabonomics, as an important part of systems biology, are emerging fields after genomics, proteomics, and transcriptomics. Through simultaneous qualitative and quantitative analyses of all small molecular (molecular mass < 1500 UDaltons) metabolites in biological fluids and organisms, the relative relationship between metabolites and physiological and pathological changes can be determined (4). This provides a promising alternative to overcome this problem and makes it possible to identify important biomarkers for diagnosing and assessing the risk of CAD development, even before patients begin to show overt symptoms (5). Oliver first proposed the concept of metabolomics in 1997 (6), i.e., to study the metabolic pathways of an organism by conducting qualitative and quantitative analyses of all metabolite compositions in the organism. The analysis technology mainly used is gas chromatography-mass spectrometry (GC/MS). Nicholson’s research group was the first to use nuclear magnetic resonance (NMR) to analyse the metabolic processes of animals and put forward the concept of metabonomics in 1999 (7), i.e., to study the dynamic changes in all metabolites of organisms under pathophysiological stimulation or gene modification at different times (8). To date, metabolomics and metabonomics have been integrated, and research techniques have included NMR, GC/MS, liquid chromatography-mass spectrometry (LC-MS), direct-injection mass spectrometry, and capillary electrophoresis–mass spectrometry (CE-MS) (9–11). In this study, we used metabolomics for the description.

With the continuous development of metabolomics, numerous CAD studies have been conducted worldwide (12, 13). However, it is difficult to systematically investigate the research situation in this field. Bibliometric analysis is widely used for the qualitative and quantitative analysis of knowledge in the literature (14), which plays an increasingly important role in developing guidelines and evaluating research trends (15). CiteSpace was developed by Professor Chaomei Chen of Drexel University, United States (14). It is a visualisation analysis software that was gradually developed under the background of bibliometrics and data visualisation. It can make a visual analysis of scientific research literature in a certain field and then discover the research hotspots and main research directions. It can also display the basic knowledge and hotspots of a certain research field by visualising and predicting evolutionary trends and research frontiers (16). VOSviewer is a bibliometric software developed by Van Eck and Waltman from Leiden University, Netherlands, to map knowledge (17). It is suitable for processing large-scale data, constructing relational networks, and visualising data analysis, which is characterised by strong graphics capabilities (18). VOSviewer and CiteSpace are the current software packages used for bibliometric analysis (14, 17), which can identify development trends and research hotspots in scientific research (19, 20). Many scholars have used these literature analysis methods in the medical field. Zhu et al. (21) analysed the links between gut microbiota and depression by using CiteSpace. Beshyah et al. (22) quantified the research contributions related to Ramadan fasting and diabetes using VOSviewer. Chen et al. (23) reported the research and identification of hotspots in mesenchymal stem cells in CVDs using CiteSpace and VOSviewer. Lim et al. (24) used VOSviewer to analyse the application of omics beyond the central dogma in coronary heart disease research. However, our study focused on the metabonomics of CAD. In addition to VOSviewer, CiteSpace was utilised for analysis, visually analysed metabolomics, and CAD. The lectures were collected from the Web of Science database in terms of authors, institutions, countries, journals, cited references, and keywords. These were used to detect the burstness of keywords to explore the hotspots and frontiers in this field and provide a reference basis and direction for the follow-up study of metabolomics with regards to CAD. To our knowledge, there is no bibliometrics research in the field of CAD with the application of both VOSviewer and CiteSpace software and specifically targeted on metabolomics.

## Materials and Methods

### Search Strategy

The relevant literature was obtained from the Science Citation Index Expanded (SCI-E) database of the Web of Science Core Collection (WOSCC) on September 16, 2021. The search formula was TS = (“cardiovascular disease*” OR “coronary artery disease*” OR “coronary disease*” OR “ischemic heart disease” OR “CHD” OR “CAD”) AND TS = (“Metabolomic*” OR “Metabonomic*” OR “Metabolome”). The search results were confined by language (English), literature type (article), and publication year (1985–2021). Ultimately, 1121 records were identified.

### Data Analysis

All records including the titles, authors, abstracts, keywords, and cited references, were downloaded and inputted to VOSviewer 1.6.16 and CiteSpace 5.7. R5 for the analysis of basic metrics. Journal Citation Reports (JCR) 2020 was used to obtain the impact factor (IF) and quartile of a journal category. VOSviewer can be used for almost all common bibliometric studies, such as collaborative relationships between authors, institutions, and countries/regions of highly co-cited references. Author co-citation analysis is used to study the research relationship between authors by analysing literature published by different authors and cited by another article. The characteristics can be visually reflected by the distance between the elements and the strength of the association. We also used this software to perform author and journal co-citation analyses. In addition, VOSviewer can classify keywords with high co-occurrence frequencies into several clusters and simultaneously colour them over time to identify research hotspots and trends. CiteSpace is a web-based Java application that focuses on finding critical points in the development of a field or domain, particularly intellectual turning points and pivotal points (25). The results are displayed in the form of visual graphs, where the nodes represent the research items. The size of a node is proportional to its frequency by type. Nodes are connected by lines of different colours and thicknesses; the darker the colour and the thicker the line, the greater the cooperation intensity between the nodes (14). The betweenness centrality (BC) metric was applied to quantify the importance of each node in the network (26). BC > 0.1 was often marked with purple circles (27). Furthermore, CiteSpace can capture keywords with strong citation bursts and construct visualisation maps for all items. Citation burst is a key indicator for identifying emerging trends (28). The parameters were set as follows: time span (2005–2021), years per slice (1), top N per slice (50), node type (chosen one at a time), and pruning (pathfinder and pruning sliced networks).

## Results

### Publication Output and Temporal Trend

**FIGURE 1:**
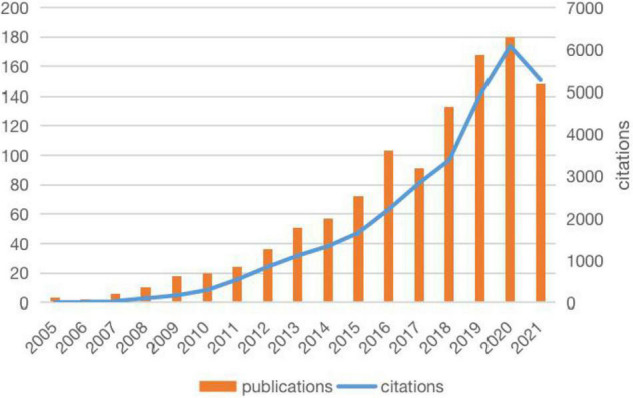
Trends in publications and citations in metabolomics and coronary artery disease research (2005–2021). The publications generally showed an upward trend, annual citations were also increased during the past decade.

The literature related to metabolomics and CAD was first published in 2005, and the publications generally showed an upward trend, except for a slight decline in 2017 (Figure 1). Annual citations have also increased over the past decade, especially from 2018 to 2020. Both the number of publications and citations reached their highest values in 2020, with 180 articles and 6083 citations, respectively. Relevant papers were continuously updated by the date of retrieval, and the average citation count of each article was 27.33. As can be seen from Table 1, The United States had the most publications, with 414 (36.931%) articles, followed by the People’s Republic of China 299 (26.673%) and England 146 (13.024%). The total number of studies conducted by the top two countries comprised more than half of the total. In terms of mean citations, the top three countries were Finland (53.64), Sweden (50.73), and Germany (49.19), indicating that these countries had a high research interest in this field. The top ten most productive institutions are shown in Table 2. The institutions with the highest number of research publications in this field are Harvard University with 100 publications (8.921%), followed by the University of California System with 57 publications (5.085%) and Brigham Women’s Hospital with 56 publications (4.996%). Harvard University also had the highest centrality, reminding us that these institutions have conducted extensive research in this field.

**TABLE 1:** Top 10 countries by publications, citations, and centrality of coronary artery disease and metabolome research.

Rank	Country	Publications	% of 1,121	Total citations	Average citations	Rank	Country	Centrality
1	USA	414	36.931	16,603	40.1	1	USA	0.30
2	People’s Republic of China	299	26.673	5,283	17.67	2	Germany	0.27
3	England	146	13.024	5,306	36.34	3	Sweden	0.17
4	Germany	91	8.118	4,476	49.19	4	Netherlands	0.17
5	Spain	85	7.583	2,076	24.42	5	England	0.17
6	Italy	67	5.977	1,947	29.06	6	Scotland	0.14
7	Finland	64	5.709	3,433	53.64	7	France	0.12
8	Netherlands	60	5.352	1,834	30.57	8	Australia	0.11
9	Sweden	55	4.906	2,790	50.73	9	Qatar	0.10
10	Canada	47	4.193	1,814	38.6	10	Denmark	0.09

**TABLE 2:** Top 10 institutions distributed by publications and centrality with regards to coronary artery disease and metabolome research.

Rank	Institution	Publications	Original Country	Institution	Centrality	Original Country
1	Harvard University	100	United States	Harvard University	0.17	United States
2	University of California System	57	United States	Imperial College London	0.11	England
3	Brigham and Women’s Hospital	56	United States	University of Copenhagen	0.1	Denmark
4	Harvard Medical School	51	United States	University of Helsinki	0.1	Finland
5	University of London	51	England	University of Michigan	0.1	United States
6	Harvard T.H. Chan School of Public Health	50	United States	German Resource control environment Hlth	0.1	Germany
7	Helmholtz Association	46	Germany	Albert Einstein Cole Hospital	0.1	United States
8	Imperial College London	45	England	General Hospital of Massachusetts	0.09	United States
9	Massachusetts Institute of Technology	44	United States	Meabolon Inc.	0.08	United States
10	Broad University	43	United States	University and Rovera University and Virgili Universit	0.08	Spain

### Distribution of Countries/Regions and Institutions

**FIGURE 2:**
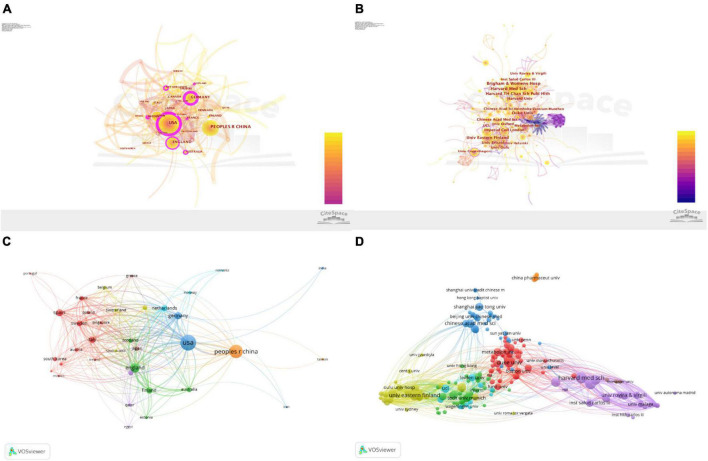
Visualisation map of the countries and institutions involved in coronary artery disease and metabolome research. Collaboration among countries/regions **(A)** and institutions **(B)** using CiteSpace. The node represents the country/region or institute. The thickness of the lines indicates the strength of the relationship. The betweenness centrality of the nodes with the purple circle is >0.1. Collaboration network of countries/regions **(C)** and institutes **(D)** using VOSviewer. Different colours mean clusters of an intimate relationship.

As shown in Figure 2A, the nodes of the United States, England, Sweden, Netherlands, France, Scotland, and Australia are marked with a purple circle, which indicates that the United States, Finland, Germany, and Sweden have a great influence on metabolomics research on CAD. Visualised knowledge mapping can provide information on influential research terms and potential collaborators, and help researchers establish collaborative relationships (29). As can be seen from Figure 2B, the nodes of the Harvard University, Duke University, Imperial College London, and University of Copenhagen are marked with a purple circle, which reminds us that these institutions have extensive research in this field. As shown in Figure 2C, different colours indicate clusters of intimate relationships, and countries with close cooperation can be subdivided into six types. The blue part: the United States showed more cooperation with Germany, Netherlands, Norway, and Romania; the brown part: in addition to having close ties with the United States, China also has close co-operations with Japan and Greece; the green part: England worked more frequently with Finland, Australia, Scotland, and Estonia; the red part: Spain worked closely with Sweden, Italy, France, South Korea, Brazil, Portugal, and Austria; the yellow part: Denmark, Belgium, Switzerland, and Saudi Arabia were more closely connected; the purple part: Canada had more ties with Qatar and Egypt. As shown in Figure 2D, the purple part: Harvard Medical School cooperated closely with the Brigham and Women’s Hospital, Harvard University, and Rovira Virgili University; the yellow part: the University of Eastern Finland worked more closely with the University of Helsinki and Imperial College London; the blue part: the Chinese Academy of Sciences had more cooperation with Shanghai Jiao Tong University, Hong Kong Baptist University, and Shanghai University of Traditional Chinese Medicine; the red part: Duke University worked with Emory University closely, and through the brown part, China Pharmaceutical University is also shown in the figure, but has no contact with other institutions.

### Distribution of Authors and Co-cited Authors

**FIGURE 3:**
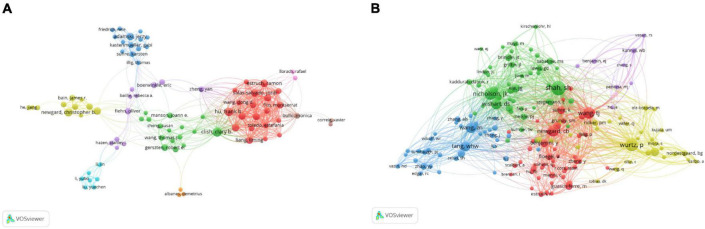
VOSviewer visualisation map of co-operation and co-citation of authors related to coronary artery disease and metabolome research. **(A)** The co-operation of network of authors. Different colours represent clusters of close cooperation. The thickness of the lines indicates the strength of the relationship. **(B)** Co-citation network of authors. Shah SH had the highest number of co-citations.

In the past 16 years, the authors ranked among the top 10 in the number of papers published on metabolomics and CAD are shown in Table 3, among which Ala-Korpela M is the most productive author from the University of Oulu Fac Med in Finland, publishing 28 articles, followed by Clish CB from the Broad Institute Cambridge in the United States (24) and Adamski J from the Helmholtz Association Inst Expt Genet in Germany (22). The top ten authors’ institutions are mostly in North America and Europe. Three of the 10 most frequently cited authors, Hazen SL (3239 citations), Tang WH (3239 citations), and Wang ZN (3162 citations), are all from the Cleveland Clinic Foundation. In addition, we noticed that five of the ten most productive authors (Ala-Korpela M, Adamski J, Newgard CB, Soininen P, Gerszten RE) were also among the most frequently cited authors, which indicated that these five researchers have high international prestige in this area. As shown in Figure 3A, different colours represent clusters of close cooperation. Clish CB cooperated closely with Gerszten RE, Wang TJ, Manson JE, and Kathryn M. In addition to being in close contact with Corella D, Hu FB is also frequently collaborating with Salas SJ, Estruch R, and so on. As shown in Figure 3B, Shah SH has the highest co-citations, followed by Nicholson JK and Wurtz P, and all their co-citations exceed 200. The above analysis suggests that they have a strong academic reputation in this area.

**TABLE 3:** Top 10 authors distributed by publications and citations.

Rank	Author	Publications	Country	Institution	Cited author	Cited frequency	Country	Institution
1	Ala-korpela, M	28	Finland	University of Oulu Fac Med	Hazen, SL	3274	United States	Cleveland Clinic Foundation Lerner Res Inst
2	Clish, CB	24	United States	Broad Institute Cambridge	Tang, WH	3274	United States	Cleveland Clinic Foundation Dept Cardiovasc Med
3	Adamski, J	22	Germany	Helmholtz Association Inst Expt Genet	Wang, ZN	3193	United States	Cleveland Clinic Foundation Lerner Res Inst
4	Hu, FB	20	United States	Harvard University Sch Publ Hlth	Allayee, H	2918	United States	Keck Sch Med USC Los Angeles
5	Kangas, AJ	19	Finland	Nightingale Hlth Ltd.	Newgard, CB	1314	United States	Duke University Med Ctr
6	Soininen, P	19	Finland	University of Oulu Inst Clin Med	Gerszten, RE	1172	United States	Beth Israel Deaconess Medical Center Div Cardiovasc Med
7	Gerszten, RE	18	United States	Beth Israel Deaconess Medical Center Div Cardiovasc Med	Ala-korpela, M	992	Finland	University of Oulu Fac Med
8	Li, Y	18	United States	Mayo Clinic Dept Cardiovasc Med	Shah, SH	968	United States	Duke University Sch Med
9	Martinez-Gonzalez, MA	17	Spain	Instituto de Salud Carlos III Ctr Invest Biomed Red Fisiopatol Obesidad and Nutr	Milburn, MV	927	United States	Metabolon Inc.
10	Newgard, CB	17	United States	Duke University Med Ctr	Adamski, J	924	Germany	Helmholtz Association Inst Expt Genet
								

### Distribution of Journals

**FIGURE 4:**
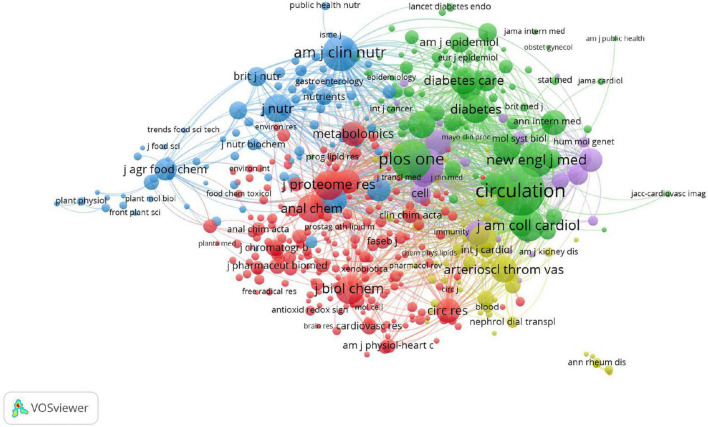
VOSviewer visualisation map of co-citation journals devoted to coronary artery disease and metabolome research. A node represents a journal; the node with a large size represents the keyword with frequent citation. The top three co-cited journals were Circulation, PLoS One, and the American Journal of Clinical Nutrition.

As shown in Table 4, the top three prolific journals were Scientific Reports (IF 4.379), PLoS One (IF 3.24), and the Journal of Proteome Research (IF 4.466). In addition, the Journal of Circulation (IF 29.69) had the highest citations, followed by PLoS One (IF 3.24) and the Journal of Proteome Research (IF 4.466). Seven of the top ten journals are also the most frequently cited, which indicates that these seven journals are highly authoritative in this field. In addition, the top three co-cited journals were Circulation (1763 citations), PLoS One (1218 citations), and the American Journal of Clinical Nutrition (956 citations) (Figure 4). Most journals listed in Table 4 were classified as Q1 or Q2, and these results indicate that the above journals have a high academic reputation in the research on metabolomics and CAD.

**TABLE 4:** Top 10 journals distributed by publications and citations.

Rank	Journal	Publications	% of 1,121	IF (JCR 2020)	JCR quartile	Cited journal	Total citations	IF (JCR 2020)	JCR quartile
1	Scientific Reports	47	4.193	4.379	Q1	Circulation	2393	29.69	Q1
2	PLoS One	42	3.747	3.24	Q2	PLoS One	1770	3.24	Q2
3	Journal of Proteome Research	37	3.301	4.466	Q1	Journal of Proteome Research	1232	4.466	Q1
4	Metabolomics	37	3.301	4.29	Q2	Metabolomics	863	4.29	Q2
5	American Journal of Clinical Nutrition	23	2.052	7.045	Q1	PLoS Genetics	704	7.045	Q1
6	Metabolites	21	1.873	4.932	Q2	Journal of The American College of Cardiology	659	29.983	Q1
7	Circulation	18	1.606	29.69	Q1	European Heart Journal	649	5.917	Q1
8	Journal of The American Heart Association	17	1.517	5.501	Q1	American Journal of Clinical Nutrition	631	24.094	Q1
9	Circulation Cardiovascular Genetics	16	1.427	4.534	Q1	Circulation Cardiovascular Genetics	599	4.534	Q1
10	Frontiers In Pharmacology	15	1.338	5.914	Q1	Circulation Research	573	4.379	Q1

### Highly Cited Reference Analysis

As shown in Table 5, “Gut flora metabolism of phosphatidylcholine promotes cardiovascular disease” by Wang et al., published in 2011, was cited the most often (2658 citations). This article predicted the risk of cardiovascular disease in a large independent clinical cohort by investigating three metabolites of the dietary lipid phosphatidylcholine and discovered the relationship between gut flora-dependent metabolism of dietary phosphatidylcholine and cardiovascular disease pathogenesis (30). The second most cited article was “Human gut microbes impact host serum metabolome and insulin sensitivity” by Pederson et al., published in 2016 (734 citations). This study discussed how the human gut microbiome impacts the serum metabolome and indicated that microbial targets might have the potential to diminish insulin resistance and reduce the incidence of common metabolic and cardiovascular disorders (32). “Phenol-Explorer: an online comprehensive database on polyphenol contents in foods” by Neveu et al., published in 2010 (33), was the third most cited article (636 citations). This study reported that the researchers developed Phenol-Explorer, which is the first attempt to systematically collect information on an essential part of the plant metabolome (polyphenols) with specific attention to food, and it should help researchers to better understand the role of phytochemicals in the technical and nutritional quality of food (33). Previous studies in this field have laid the foundation for further clinical and experimental research in CAD. The authors of these articles’ academic value in this field is highly recognised. However, it should be noted that articles published earlier are more likely to be cited than articles published later, and we should consider the influence of time as a confounding factor in the cited literature analysis.

**TABLE 5:** Top 10 cited literature.

Rank	Citations	Title	Author	Journal	Year	DOI
1	2658	Gut flora metabolism of phosphatidylcholine promotes cardiovascular disease	Wang et al. (30)	Nature	2011	10.1038/nature09922
2	734	Human gut microbes impact host serum metabolome and insulin sensitivity	Pedersen et al. (32)	Nature	2016	10.1038/nature18646
3	636	Phenol-Explorer: an online comprehensive database on polyphenol contents in foods	Neveu et al. (33)	Database(Oxford)	2010	10.1093/database/bap024
4	452	Gut microbiota dysbiosis contributes to the development of hypertension	Li et al. (101)	Microbiome	2017	10.1186/s40168-016-0222-x
5	442	Genetics Meets Metabolomics: A Genome-Wide Association Study of Metabolite Profiles in Human Serum	Gieger et al. (102)	PLoS Genetics	2008	10.1371/journal.pgen.1000282
6	395	Alpha-Hydroxybutyrate Is an Early Biomarker of Insulin Resistance and Glucose Intolerance in a Non-diabetic Population	Gall et al. (103)	PLoS One	2010	10.1371/journal.pone.0010883
7	367	Paraoxonase-1 is a major determinant of clopidogrel efficacy	Bouman et al. (104)	Nature Medicine	2011	10.1038/nm.2281
8	344	Metabolite Profiling Identifies Pathways Associated With Metabolic Risk in Humans	Cheng et al. (105)	Circulation	2012	10.1161/circulationaha.111.067827
9	335	Metabolomic identification of novel biomarkers of myocardial ischemia	Sabatine et al. (60)	Circulation	2005	10.1161/circulationaha.105.569137
10	320	Prognostic value of choline and betaine depends on intestinal microbiota-generated metabolite trimethylamine-*N*-oxide	Wang et al. (74)	European Heart Journal	2014	10.1093/eurheartj/ehu002

### Keyword Analysis

#### Keyword Co-occurrence

Keywords reflect the core of the content of the article and can be used to analyse the frontiers of knowledge related to the research field. As shown in Table 6, in addition to metabolomics (509) and cardiovascular disease (322), the keywords used with a high frequency of occurrence were risk (224), biomarkers (145), insulin resistance (129), and atherosclerosis (110). Keywords with high centrality were adipose tissue, proteomics, artery disease, C-reactive protein, coronary artery disease, metabonomics, and atherosclerosis.

**TABLE 6:** Top 20 keywords with the most occurrences and total link strength.

Rank	Keywords	Occurrences	Total link strength	Rank	Keywords	Occurrences	Total link strength
1	metabolomics	509	3711	11	coronary artery disease	98	786
2	cardiovascular disease	322	2299	12	oxidative stress	77	536
3	risk	224	1753	13	identification	66	502
4	biomarkers	145	1159	14	mass spectrometry	64	444
5	insulin resistance	129	1017	15	gut microbiota	59	464
6	atherosclerosis	110	897	16	mortality	54	419
7	plasm	107	827	17	serum	54	406
8	obesity	98	786	18	lipidomics	53	420
9	inflammation	92	689	19	epidemiology	53	397
10	association	90	687	20	cholesterol	52	409

**FIGURE 5:**
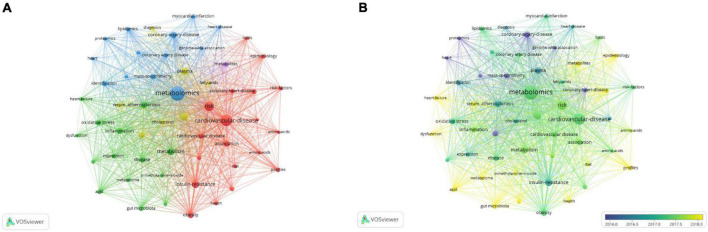
Analysis of keywords in studies related to metabolomics and coronary artery disease. **(A)** VOSviewer visualisation map of co-occurring keywords. The node represents keywords, and a larger size represents the keyword that appeared at a higher frequency. The thickness of the lines indicates the strength of the relationship. The colours of nodes are used to indicate different clusters. **(B)** VOSviewer overlay visualisation map of co-occurring keywords. The colour of the node indicates the time when the topic word appears, and the emergence time of each keyword is the average value of the project in which it was established.

As shown in Figure 5A, in the keyword co-occurrence network map, the thicker the connection between the nodes is, the more frequently the two keywords appeared together. The keywords mainly formed four clusters, representing the four major research directions in the field. The blue cluster (cluster #1) was dominated by lipidomics, proteomics, genome-wide association, identification, and mass spectrometry, which were mainly related to the description of the research methods. Lipidomics has been used for research on CAD clinical endpoint events (34–36), left ventricular remodelling (37–39), and so on. The combined use of proteomics and metabolomics provides a way to better understand the mechanisms of CAD (40). Genome-wide association research has explored related explorations in the aspects of genetic variation (41–43). The most typical of these is the mutation that occurs in the gene encoding apolipoprotein E. In addition, the genetic variation in the *FADS* gene family (*FADS1*, *FADS2*, and *FADS3*) responsible for encoding exogenous and endogenous lipid desaturases has been largely reported to be related to the concentration of circulating polyunsaturated fatty acids (44–46). Systems biology addresses the problems posed by the complex organisation of biological processes (47). Multi-omics platforms and front-facing technical methods have constituted an important direction in systems biology research. Mass spectrometry-related technologies mainly include (11): LC-MS, GC-MS, CE-MS, and chromatography-nuclear magnetism-mass spectrometry. The red cluster (cluster #2) was mainly composed of insulin resistance, association, epidemiology, profiles, amino acid, diet, obesity, and risk factors for CAD. Insulin resistance is the most fundamental pathophysiological basis of type 2 diabetes (48), it can exacerbate atherosclerosis (49), and is directly related to CAD. By applying metabolomics to large-scale epidemiological studies, key biomarkers and novel pathways are rapidly being discovered (50). Metabolic Profiling of amino acid, fatty acid, and lipoprotein is also included in the red cluster. Studies have found that fatty acids are associated with cardiac insufficiency (51), progression of coronary atherosclerosis (52), and sudden death (53). Evidence has shown that high levels of the branched-chain amino acids are associated with cardiovascular mortality and acute heart failure after revascularization (54) and are significantly correlated with the occurrence and development of CAD (55). The green cluster (cluster #3) focused on aetiology and pathogenesis research, and was mainly composed of atherosclerosis, inflammation, oxidative stress, gut microbiota, trimethylamine *N*-oxide, and dysfunction. Inflammation plays an important role in the development and progression of CAD. Related research mainly focuses on high-sensitivity C-reactive protein (hs-CRP), interleukin-6 (IL-6), and tumour necrosis factor-α (TNF-α), and so on, among which hs-CRP is an independent predictor of coronary artery inflammation (56). Studies have found that the intestinal microbiota-dependent metabolite produced by choline and phosphatidylcholine TMAO can predict early CAD and atherosclerosis (30, 57–59). Microbial metabolites provide new research directions for the diagnosis and treatment of atherosclerotic heart disease and have become a research hotspot in CAD metabolomics. The yellow cluster (cluster #4) was mainly composed of serum, cholesterol, plasma, diagnosis. Metabolomics technology is used to study the metabolome of various bodily fluids, such as plasma (60) and urine (61), perform qualitative and quantitative analyses of metabolites, and assist in the diagnosis or evaluation of CAD and its severity.

As shown in the keyword VOSviewer overlay visualisation map (Figure 5B), the colour of the node indicates the time at which the topic word appears, and the emergence time of each keyword is the average value of the project in which it was established. It can be seen from the figure that keywords such as proteomics, mass spectrometry, genome-wide association, and plasma are the hot spots of early research. Current research hotpots include profiles, epidemiology, serum, acid, heart failure, dysfunction, metabolites, gut microbiota, and trimethylamine-*N*-oxide.

#### Burstness of Keywords

**FIGURE 6:**
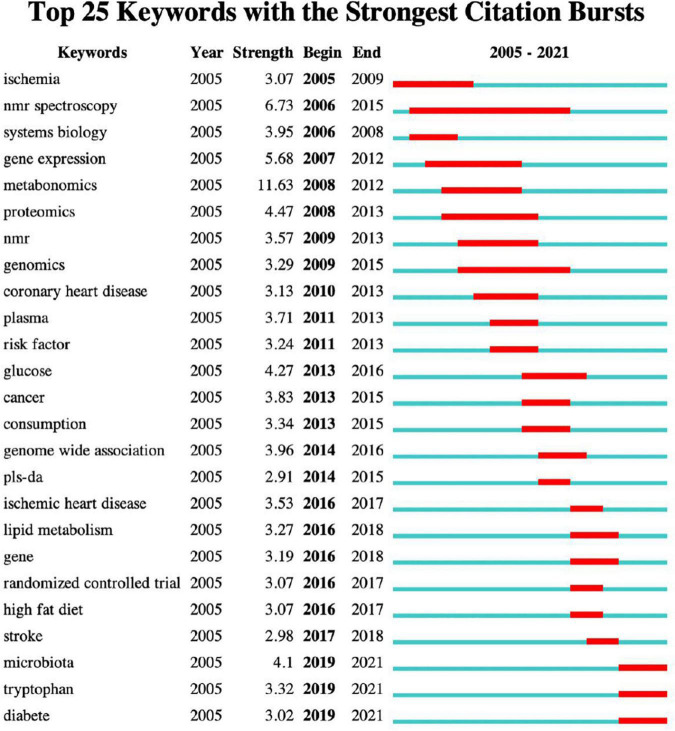
Keywords with the strongest citation bursts. The blue line indicates the timeline, and the red segment on it indicates the duration of the burst.

CiteSpace was used to perform burst detection of keywords (Figure 6). Keywords burstness can reflect emerging academic trends and new topics, predict frontier research directions, and reveal potential hotspots in a field. The timeline is depicted as a blue line, whereas burst detection is shown as a red segment on the blue timeline, which indicates the start year, end year, and duration of the burst. By analysing the keywords in this field, 25 prominent words were obtained. “Metabonomics” showed the strongest burst strength, followed by “NMR spectrometry” and “gene expression.” Currently, “NMR spectrometry” has the longest duration. However, “high-fat,” “diet,” “stroke,” and “randomised controlled trial” persisted for a shorter period. We were particularly interested in keywords that started to burst from 2019, including “microbiota” (burst strength 4.1), “tryptophan” (burst strength 3.32), and “diabetes” (burst strength 3.02), which are the current research frontiers in this field and are currently within the burst period.

## Discussion

This study used VOSviewer 1.6.16 and CiteSpace 5.7. R5 to analyse 1121 articles in the Web of Science database on metabolomics in CAD published from 2005 to 2021. Temporal and spatial distributions, author contributions, core literature, research hotspots, and frontiers were evaluated. We used keyword co-occurrence analysis to identify research hotspots in each period and determine the core evolutionary path of the theme. We identified the current research frontiers of metabolomics in the field of CAD. The main conclusion are as follows.

### General Distribution

The literature related to metabolomics and CAD included in the Web of Science database was first published in 2005, and the annual publication output has been steadily increasing since then. Citation analysis can provide important information about the frequency of literature use and citations, which can objectively measure the frequency of researchers’ published works and provide priority information support for research progress (62, 63). The number of publications and citations reached its highest value in 2020, which may be attributed to the worldwide increase in CAD patients, and the research on metabolomics and CAD is receiving increasing attention. Relevant papers were continuously updated by the date of retrieval, indicating that research in this field was still in the stage of rapid development.

The United States had the highest number of publications, total citations, and centrality, which was also much higher than that of other countries. Although Sweden ranked ninth in the number of publications, its centrality ranked third and it had the second most cited frequency, suggesting a very high quality of its publications. Although the number of published articles in China ranks second, showing that China has certain strength in this field, the centrality is 0.04 (Table 1), cooperation with other countries/regions is also less (Figure 2C), and there is no Chinese institution in the list of the top ten institutions (Table 2). Although China Pharmaceutical University is also shown in the figure, it has no contact with other institutions, which indicates that China still has space to improve in strengthening exchanges with other countries and institutions. Among the top ten institutions, most were from America; the centrality of Harvard University, Imperial College London, University of Copenhagen, University of Helsinki, University of Michigan, German Resource Control Environment Health, and Albert Einstein Cole Hospital were more than 0.1, which are marked with a purple circle (Figure 2B), suggesting that the United States and the institutes from the United States are the leading driving forces in this research field. This maximised its geographical advantages and further strengthened its academic influence on metabolomics in CAD research.

Additionally, our results demonstrated that Ala-Korpela M was the most productive author, who used clinical epidemiology and metabolomics methods such as ^1^H NMR to study in this field (64–66). Moreover, Hazen SL was the author with the highest number of citations, who utilised metabonomics technology to study risk factors (67), mechanisms, and treatment of CAD (68, 69). The author with the highest number of co-citations was Shah SH, who mainly studied metabolomic profiling related to cardiovascular diseases (70, 71), and utilised multi-omics platforms to elucidate novel genetic markers and mechanisms of disease (72). These experts are active in this field and have more research results on the relationship between metabolomics and CAD, which plays an important guiding role. We also noticed that the top three frequently cited authors (Hazen SL, Tang WH, and Wang ZN) are all from the Cleveland Clinic Foundation (Table 3), suggesting that the Cleveland Clinic Foundation is an important institution in the research field.

JCR 2020 was used to obtain the impact factor (IF) and quartile (Q) of each journal category (73). The Journal Citation Reports division divides journals in the same discipline into four equal parts, with the top 25% being Q1 and 25–50% being Q2. The most productive journals were classified as Q1 or Q2. Although Scientific Reports (IF 4.193 Q1), PLoS One (IF 3.24, Q2), and the Journal of Proteome Research (IF 4.466, Q1) were the top three productive journals, their IF values were less than 5. This indicates that improving research quality while increasing output may enhance academic influence. Among the most commonly cited journals, Circulation (IF29.69, Q1) had the highest IF, which suggests that it published journals with excellent academic quality widely recognised by the majority of peers.

### Hotspots and Frontiers

The analysis of keywords in the literature can explore research hotspots and core content in this field. “risk,” “biomarkers,” “insulin resistance,” “atherosclerosis,” “obesity,” “inflammation,” “Oxidative stress,” “mass spectrometry,” “gut microbiota,” and so on are the main keywords of the current study (Table 6). Among them, we can see the research hotspots in recent years: (i) Research on aetiology and pathogenesis in this field, such as insulin resistance (34, 74), atherosclerosis (37), inflammation (41), dysfunction (39); the studies on these factors have been described in the keyword co-occurrence section. (ii) Microbiota and metabolites. Studies have shown that the gut microbiome is a potential diagnostic and pharmacological target for the prevention and treatment of CAD (75). Microbiota metabolites play important roles in cardiometabolic health. TMAO as one of the metabolites has been observed to be closely related to CAD (76, 77). Several epidemiological studies have shown the prognostic value of TMAO plasma levels, which can predict future cardiovascular events (78–80). The mechanism may be related to the upregulation of scavenger receptor expression in macrophages (30) and an increase in the expression of cellular HSPs (81) and pro-inflammatory cytokines (82, 83). However, the realisation of TMAO as a target to prevent and treat CAD requires more in-depth research in the future. (iii) Epidemiological studies have investigated the roles of various metabolic pathways related to CAD. For example, lipid metabolic pathways (84–86), and other metabolic pathways, such as NMR-based metabolic fingerprints are associated with CAD (87, 88). Based on the results of the keyword burst analysis, we speculate that the study of the tryptophan (Trp) metabolic pathway regulated by the gut microbiome will become a new academic trend in the fields of metabolomics and CAD. Trp is an essential amino acid in the human body that can be metabolised into a variety of molecules, such as indole and its derivatives, by microorganisms in the gut (89). In the Trp metabolic pathway, indoleamine 2,3-dioxygenase (IDO)1 promotes arteriosclerosis progression. The level of the metabolite kynurenic acid (Kna) (90) produced by the action of IDO1, can predict death and recurrent myocardial infarction in patients admitted to the hospital because of acute myocardial infarction (91). Circulating kynurenine (Kyn) and Kyn-derived metabolites are associated with cardiovascular risk factors (92, 93) and the poor prognosis in patients with CAD (94–96). Studies have also shown that metabolites derived from the gut microbiota, such as butyrate, negatively regulate IDO expression by intestinal epithelial cells (97) and have a potential role in regulating Trp metabolism. Although several bacteria that produce catabolites of Trp have been identified, the major contributors to the catabolism of Trp are still unknown. Therefore, it is an important direction for future development to search for intestinal flora interacting with CAD and to use metabolomics techniques to identify markers in the Trp metabolic pathway. In addition, determining the exact role of Trp metabolites in host physiology and adopting interventions based on the gut microbiota as treatment targets may become a new approach for the treatment and prevention of CAD in the future.

### Limitations

To date, this is the first bibliometric analysis in which CiteSpace and VOSviewer were both applied to simultaneously survey the hotspots and cutting-edge metabolomics in the field of CAD during the past decade, which enables our research results to be more accurate and objective. However, the present study had some limitations. First, all data were extracted only from the WOSCC database. However, the WOSCC database is updated continuously and dynamically, which can provide the most authoritative, relevant, and comprehensive information (98–100). It is considered to be the most prominent database of scientific publications on many research topics. Second, the amount of literature related to this field is huge, and with the rapid updating of hot topics and research frontiers in metabolomics and CAD, we may have missed some research hotspots.

## Conclusion

Studies on metabolomics in CAD have a great deal of research value and broad application prospects. With the help of CiteSpace and VOSviewer, we have furthered our understanding of this complex, multifactorial disease. To determine the exact role of metabolites in host physiological and pathological processes. Intervention and treatment of the gut microbiome, especially the regulation of gut microbiome on tryptophan metabolism, will be the research trend in the future. The results of this study are expected to promote further research and development in this field and provide a reference and guidance for the prevention and control of CAD.

## Data Availability Statement

The original contributions presented in the study are included in the article/supplementary material, further inquiries can be directed to the corresponding authors.

## Author Contributions

NY, LZ, and BL designed the study. NY collected and verified the data. NY and BL performed software analyses. NY and RW drafted the first version of this manuscript. LZ and BL revised the manuscript. All authors have read and approved the final manuscript.

## Conflict of Interest

The authors declare that the research was conducted in the absence of any commercial or financial relationships that could be construed as a potential conflict of interest.

## Publisher’s Note

All claims expressed in this article are solely those of the authors and do not necessarily represent those of their affiliated organizations, or those of the publisher, the editors and the reviewers. Any product that may be evaluated in this article, or claim that may be made by its manufacturer, is not guaranteed or endorsed by the publisher.

## Abbreviations

CVD: cardiovascular disease
CAD: coronary artery disease
CHD: coronary heart disease
WOSCC: Web of Science Core Collection
SCI-E: Science Citation Index Expanded
JCR: Journal Citation Reports
IF: impact factor
BC: betweenness centrality
FADS: fatty acid desaturases
LC-MS: liquid chromatography-mass spectrometry
GC-MS: gas chromatography-mass spectrometry
CE-MS: capillary electrophoresis-mass spectrometry
hs-CRP: high-sensitivity C-reactive protein
IL-6: interleukin-6
TNF- α: tumour necrosis factor- α
TMAO: trimethylamine-*N*-oxide
NMR: nuclear magnetism
Trp: tryptophan
IDO: indoleamine 2,3-dioxygenase
MI: myocardial infarction
Kna: kynurenic acid
Kyn: kynurenine.
