# Corrigendum: Bibliometric and Visual Analysis on Metabolomics in Coronary Artery Disease Research

**DOI:** 10.3389/fcvm.2022.937508

**Published:** 2022-06-28

**Authors:** Ning Yu, Ruirui Wang, Baocheng Liu, Lei Zhang

**Affiliations:** Shanghai Innovation Center of TCM Health Service, Shanghai University of Traditional Chinese Medicine, Shanghai, China

**Keywords:** VOSviewer, CiteSpace, metabolomics, coronary artery disease, visual analytics

In the published article, there was an error in Table 1 as published. The published version of Table 1 was a duplicated version of Table 6. The corrected Table 1 and its caption appear below.

**Table 1:** Top 10 countries by publications, citations, and centrality of coronary artery disease and metabolome research.

**Rank**	**Country**	**Publications**	**% of 1,121**	**Total citations**	**Average citations**	**Rank**	**Country**	**Centrality**
1	USA	414	36.931	16,603	40.1	1	USA	0.30
2	People's Republic of China	299	26.673	5,283	17.67	2	Germany	0.27
3	England	146	13.024	5,306	36.34	3	Sweden	0.17
4	Germany	91	8.118	4,476	49.19	4	Netherlands	0.17
5	Spain	85	7.583	2,076	24.42	5	England	0.17
6	Italy	67	5.977	1,947	29.06	6	Scotland	0.14
7	Finland	64	5.709	3,433	53.64	7	France	0.12
8	Netherlands	60	5.352	1,834	30.57	8	Australia	0.11
9	Sweden	55	4.906	2,790	50.73	9	Qatar	0.10
10	Canada	47	4.193	1,814	38.6	10	Denmark	0.09

The authors apologize for this error and state that this does not change the scientific conclusions of the article in any way. The original article has been updated.

## Publisher's Note

All claims expressed in this article are solely those of the authors and do not necessarily represent those of their affiliated organizations, or those of the publisher, the editors and the reviewers. Any product that may be evaluated in this article, or claim that may be made by its manufacturer, is not guaranteed or endorsed by the publisher.

